# Comment on: Benchmarking tocilizumab use for giant cell arteritis

**DOI:** 10.1093/rap/rkac069

**Published:** 2022-08-23

**Authors:** Shalini Janagan, Catherine Guly, Sarah Skeoch, Joanna C Robson

**Affiliations:** Department of Rheumatology, University Hospitals Bristol and Weston NHS Foundation Trust, Bristol, UK; Bristol Eye Hospital, University Hospitals Bristol and Weston NHS Foundation Trust, Bristol, UK; Department of Rheumatology, Royal National Hospital for Rheumatic Diseases, Royal United Hospitals, Bath, UK; Department of Rheumatology, University Hospitals Bristol and Weston NHS Foundation Trust, Bristol, UK; Rheumatology Research, Faculty of Health and Applied Science, University of the West of England, Bristol, UK

Dear Editor, It is with great interest that we read the editorial on tocilizumab (TCZ) use in GCA published on 9 May 2022 by Conway *et al.* [1]. We write to share some of our data to add weight to the views expressed, particularly in relationship to use of TCZ beyond 1 year in refractory cases with visual involvement.

In line with NHS England’s policy that all cases of refractory or relapsing GCA being considered for TCZ should be discussed regionally, a peer-to-peer Bristol and Bath TCZ multidisciplinary meeting has been held monthly since November 2018, with patients referred from rheumatology and ophthalmology sites across the region. Thirty-eight cases have been discussed between November 2018 and September 2021, with 31 being approved for TCZ use. The mean age of approved cases was 74 years, with three-quarters (74.2%) being female.

Of these, 11 had refractory GCA and 20 had relapsing GCA. Most patients (77.4%) had cranial GCA, with 48.4% having large vessel vasculitis. About 45% (*n* = 14) had visual involvement, with ∼25.8% having visual loss compared with 24% with ocular symptoms reported in a Scottish cohort [2]. All patients had been on glucocorticoids, with the average time to referral being 591 days. Among them, 19.4% had hypertension, cataract progression, weight gain or osteoporosis; 16.1% had diabetes, neuropsychiatric symptoms or sleep disturbances attributed to glucocorticoid use.

On comparing patients with visual involvement *vs* those without, it was seen that those with visual involvement had presented with headache, jaw pain and scalp tenderness more commonly than large vessel vasculitis-GCA (73.8 *vs* 52.9%). They were referred to the multidisciplinary meeting earlier (478.2 *vs* 648.1 days) and were on higher doses of glucocorticoids at the time of referral (71.4 *vs* 47.1% on ≥40 mg).

In December 2021, a follow-up audit revealed that 14 of 31 patients had completed ≥12 months of TCZ; 5 of these had had an extension under coronavirus disease 2019 (COVID-19) exceptional guidance (mean duration of 5.2 months). Of the remaining 17, 3 patients had stopped early [1 death, 1 moved away and 1 owing to adverse effects (headache and gastrointestinal side effects)], 4 had not started treatment and 10 had not completed 12 months.

Adverse events in the 14 patients at 12 months included: liver abnormalities (2 of 14; 14.3%), neutropenia (2 of 14; 14.3%), thrombocytopenia (1 of 14; 7.1%), soft tissue infections (3 of 14; 21.4%), urinary tract infections (1 of 14; 7.1%) and lipid derangement (4 of 14 28.6%). One patient was admitted with chest pain but with normal investigations. One case of GCA relapse occurred on TCZ (mild headache and raised inflammatory markers, which settled on increase in prednisolone). After 12 months, the mean prednisolone dose was 3 mg (range 0–15 mg; median 1 mg).

Our data show that patients on TCZ were able to reduce the dose of glucocorticoids and associated side effects significantly and that clinicians and patients chose to continue TCZ beyond 12 months during the COVID-19 pandemic. There was a low incidence of GCA relapse on TCZ, and visual symptoms were not seen as part of any flare. Data from other studies also show similar outcomes [3]. This supports the use of TCZ beyond 12 months; abrupt withdrawal of treatment can precipitate flare-up of GCA, with significant morbidity and mortality from the disease and glucocorticoids [3]. Biologic therapies for other rheumatic diseases are funded under National Institute for Health and Care Excellence guidance until the patient and clinician decide that it is appropriate to stop. Despite this, recent new guidance from NHS England is that the policy of 1 year only (which had been extended on compassionate grounds during the COVID-19 pandemic), has now returned to a strict 1 year only treatment period, with no potential to retreat even if serious visual relapses occur.

We support the stance of EULAR in offering TCZ for patients with relapsing/refractory disease or a high risk of developing complications with glucocorticoids, with the duration of treatment decided on an individual basis [4]. Continuing TCZ beyond 12 months might prevent GCA relapse and associated morbidity, particularly in those with visual involvement, in whom relapsing disease can cause irreversible blindness and have a significant impact on function and health-related quality of life [5, 6].

## Data Availability

The data underlying this article will be shared on reasonable request to the corresponding author.
